# Attention-guided and MIL-constrained CycleGAN for high-fidelity virtual p16 and Ki-67 staining

**DOI:** 10.3389/fmedt.2026.1783365

**Published:** 2026-05-29

**Authors:** Xinli Lei, Yuefeng Xie, Andi Duan, Yuhang Wu, Linhong Liao, Xi Wang

**Affiliations:** 1 Department of Pathology, Maternal and Child Health Hospital of Ganzhou City, Ganzhou, Jiangxi, China; 2 School of Electrical and Information Engineering, Tianjin University, Tianjin, China

**Keywords:** attention mechanism, cervical intraepithelial neoplasia, computational pathology, CycleGAN, H&E-to-IHC translation, Ki-67, p16, virtual immunohistochemistry

## Abstract

Cervical intraepithelial neoplasia (CIN) grading relies on both morphological assessment from hematoxylin and eosin (H&E) slides and biomarker expression from immunohistochemistry (IHC), such as p16 and Ki-67. However, conventional IHC staining is time-consuming, costly, and requires additional tissue sections. To address these limitations, we propose an attention-guided and multi-instance learning (MIL) constrained CycleGAN framework for high-fidelity virtual IHC staining generation from H&E images. To better utilize the correspondence between H&E and IHC slides, we adopt a weakly paired training strategy. Specifically, whole-slide image registration is first applied to align H&E and corresponding IHC slides, followed by patch-level pairing for model training. Considering the inherent spatial misalignment between adjacent tissue sections, the proposed framework integrates attention-guided constraints and MIL-based supervision to enhance structural consistency and biomarker distribution reliability. In addition to quantitative image-level and diagnostic evaluations, we further conduct an expert-based clinical assessment. Two experienced pathologists independently evaluated CIN grading using H&E slides with and without virtual staining. The results demonstrate that the inclusion of virtual IHC improves diagnostic performance, supporting the clinical utility of the proposed method. To evaluate generalization capability, we additionally validate the model on an external dataset (ACROBAT) under cross-domain conditions. This experiment is designed to assess robustness rather than direct task alignment, providing supplementary evidence of model transferability. We also acknowledge the potential risk of false-positive biomarker synthesis in virtual staining. To address this, we incorporate statistical consistency constraints and provide quantitative evaluation, including diagnostic consistency and expert assessment, which together support the biological plausibility of the generated staining patterns. Overall, the proposed framework provides a practical and clinically relevant solution for virtual IHC generation, achieving superior performance across key evaluation metrics including accuracy (ACC), area under the curve (AUC), and kappa scores, while also demonstrating improved diagnostic support and cross-domain generalization capability.

## 1 Introduction

Cervical cancer remains one of the most common malignancies affecting women worldwide and represents a major global health burden. According to the International Agency for Research on Cancer (IARC), approximately 661,000 new cervical cancer cases and 348,000 related deaths were reported globally in 2022, with significantly higher mortality rates in regions with limited healthcare resources (1). Persistent infection with high-risk human papillomavirus (HR-HPV) is the primary etiological factor leading to cervical carcinogenesis. This infection induces a spectrum of pathological changes in the cervical squamous epithelium collectively referred to as squamous intraepithelial lesions (SIL) (2). Clinically, SIL is categorized into low-grade (LSIL) and high-grade (HSIL) lesions, corresponding to CIN1 and CIN2/3, respectively (3). Accurate differentiation between these categories is critical for determining appropriate clinical management strategies, including surveillance, intervention, or surgical treatment (4, 5). Histopathological examination of hematoxylin–eosin (H&E) stained sections remains the gold standard for diagnosing cervical epithelial lesions. However, the morphological spectrum of CIN lesions is continuous and heterogeneous, which often leads to overlapping histological features among different grades. Variations in nuclear atypia, mitotic activity, and epithelial stratification can introduce subjectivity in pathological interpretation (6). Previous studies have reported considerable inter-observer variability among pathologists, particularly in the diagnosis of CIN2 lesions (7). Such diagnostic inconsistencies may lead to both underdiagnosis and overtreatment in clinical practice (8).

To improve diagnostic accuracy and reproducibility, immunohistochemistry (IHC) markers are widely used as auxiliary tools in cervical lesion evaluation (9, 10). Among them, p16 and Ki-67 are the most commonly adopted biomarkers, and their combined staining patterns provide important diagnostic cues for distinguishing HSIL from LSIL (11). However, conventional IHC workflows require additional laboratory procedures, including staining, antibody incubation, and signal development, making them time-consuming, costly, and dependent on specialized infrastructure and expertise (12, 13). These limitations restrict their accessibility, particularly in primary healthcare settings and resource-limited regions, and highlight the need for scalable computational alternatives that can infer biomarker-related information directly from routine H&E slides (14).

To address these limitations, recent advances in digital pathology and artificial intelligence have enabled computational approaches for extracting diagnostic information directly from H&E images (15, 16). In particular, generative models have enabled the emergence of virtual staining, a technique that computationally synthesizes images resembling specific histological or immunohistochemical stains without performing additional laboratory procedures (17). Using deep learning frameworks such as generative adversarial networks (GANs) or diffusion models, virtual staining methods can translate H&E images into representations resembling IHC staining patterns (18, 19). Among these approaches, CycleGAN has become one of the most widely used frameworks for unpaired image-to-image translation in histopathology due to its cycle-consistency mechanism (20). Several studies have applied CycleGAN-based models to simulate histological staining patterns or generate virtual biomarker images. More recently, diffusion-based generative models and multi-domain translation architectures have also been explored to improve the diversity and realism of virtual staining outputs. Although these approaches demonstrate promising results in generating visually realistic staining patterns, most existing studies primarily treat virtual IHC generation as a style-transfer problem, focusing on color appearance and pixel-level similarity. However, pathological diagnosis relies not only on staining appearance but also on complex morphological and spatial patterns related to lesion biology. Without explicit pathology-oriented constraints, generative models may produce images that appear visually plausible but fail to preserve diagnostically relevant structural or semantic information. In particular, lesion-level patterns such as epithelial stratification, basal layer expansion, and proliferative distribution are essential for interpreting p16 and Ki-67 expression in CIN grading.

Building upon these observations, this study proposes a pathology-oriented virtual staining framework based on an enhanced CycleGAN architecture. The proposed method introduces three key components designed to incorporate diagnostic semantics into the generative process. First, a Plain–Global Attention Block is integrated into the generator to capture both fine-grained nuclear morphology and long-range epithelial structural patterns. Second, a multi-instance learning (MIL)–based auxiliary classification branch is employed to enforce lesion-level semantic consistency during training. Third, an attention-guided loss reweighting mechanism is introduced to emphasize diagnostically informative regions during optimization. By combining structural attention modeling with lesion-aware semantic supervision, the proposed framework aims to generate virtual p16 and Ki-67 staining images that better preserve clinically relevant morphological patterns and support reliable CIN assessment.

## 2 Related work

### 2.1 Computational pathology

In recent years, Computational Pathology has made significant progress in intelligent diagnosis. With the popularization of full-slice scanning technology and the optimization of deep learning models, researchers have gradually moved from early morphological classification tasks to clinically interpretable assisted decision-making systems (16). In classification and grading tasks, frameworks based on Vision Transformer (ViT) and multi-instance learning (MIL) have become mainstream. Chen et al. (21) proposed a hierarchical image pyramid converter architecture that achieves accurate slide prediction for breast, kidney, and lung cancer subtype classification tasks using only slicing labels without pixel-level labeling. Similarly, Juyal et al. proposed a joint training framework combining supervised contrastive learning and multi-instance learning, which significantly improved the classification performance of the model for subtypes of non-small cell lung cancer and renal cell carcinoma in scenarios with label imbalance and out-of-distribution by decoupling feature learning and classifier optimization. Wang et al. (22) developed an AI cervical cancer screening system based on a two-stage AI model, which achieved high-precision automatic interpretation of cervical cytological grading by integrating cell detection and whole glass slide image classification. These models indicated that the tissue structure information contained in H&E images was sufficient to support high-level pathological reasoning.

These previous studies demonstrate a correlation between routine H&E morphology and underlying molecular and spatial phenotypes. And this inherent link can be effectively leveraged to assist in clinical diagnosis. Recent research has increasingly combined H&E whole-slide images (WSIs) with biomarker/spatial prediction to actively guide adjuvant therapy. For instance, Kather et al. (23) established a standard end-to-end weakly supervised workflow capable of directly predicting clinically actionable genetic alterations from unannotated WSIs. To predict therapeutic efficacy, the DeepPT framework (24) estimates pan-cancer treatment responses by imputing whole-transcriptome expression directly from histopathology images, guiding targeted and immune therapies without the need for molecular sequencing. Similarly, in multi-modal applications, the PATH-ORACLE model (25) leverages AI-derived histopathological features to significantly improve recurrence prediction in stage I lung adenocarcinoma, effectively identifying high-risk patients who stand to benefit most from targeted adjuvant interventions.

On the other hand, as a prevailing weakly supervised paradigm for WSI analysis, recent advancements have expanded MIL’s capability to derive fine-grained spatial localization directly from weak slide-level labels. For instance, Gao et al. (26) have demonstrated mathematically and empirically that attention-based MIL frameworks can precisely identify morphological subregions and achieve accurate spatial quantification using only slide-level labels. Furthermore, X-SPATIO (27) utilizes MIL to capture complex morpho-molecular associations, successfully generating spatially resolved expression maps for targeted biomarkers directly from routine H&E images under weakly supervised settings.

These developments collectively indicate that slide-level weak labels inherently contain supervisory signals to guide the fine-grained localization of molecular phenotypes. By capturing the intrinsic correlation between bag-level macro-pathology and instance-level micro-expression, MIL can provide a robust foundation for dense prediction tasks. Consequently, this theoretical basis motivates our proposed approach to leverage MIL as a powerful auxiliary mechanism.

### 2.2 Style transfer

Style transfer, as an important paradigm for image-to-image translation, has shown great potential in medical image generation tasks in recent years. The core idea is to decouple the structural content of an image from styles such as color and texture, and to achieve cross-domain transformation through deep models (28). In the field of histopathology, traditional paired translation methods (such as Pix2Pix) are limited by their reliance on strictly aligned training data. CycleGAN (20) is widely used in establishing mapping relationships between H&E and specific staining due to its cyclic consistency mechanism. Rivenson et al. (29) proposed a deep learning framework based on generative adversarial networks that can transform autofluorescence images of unlabeled tissues into virtual stained images comparable to H&E, Masson tricolor, and Jones staining, enabling histological imaging without actual staining. However, CycleGAN has the problem of strong output determinism and lack of diversity, making it difficult to simulate the minute variations in real staining (30).

To enhance generative diversity and controllability, researchers turned to multi-domain style transfer models. StarGANv2 (31) and TUNIT (32) can support the generation of multiple target styles through a single network, and can control the output characteristics through latent vectors. Scalbert et al. (33) first applied StarGANv2 to H&E staining enhancement, verifying its potential in test-time enhancement and significantly improving the robustness of downstream classification models. However, the method requires multi-domain labeled samples for training and is difficult to apply in unlabeled scenarios. More notable is CycleGan-Turbo (34), a lightweight CycleGAN variant that combines diffusion priori to achieve high-quality, diverse image transformations while maintaining structural fidelity.

With the development of diffusion-based probabilistic modeling, recent studies have begun to explore novel I2I frameworks grounded in these advanced generative paradigms. For instance, DiffI2I (35) explores efficient architectural designs tailored specifically for I2I tasks, significantly reducing the iterative denoising burden while preventing artifact generation. From a probabilistic perspective, research on Diffusion Bridge Models (36) constructs a robust framework that establishes direct transport mappings from one conditional data distribution to another, effectively bypassing the standard Gaussian noise prior to enhance translation diversity. Alternatively, other works have investigated novel condition embedding mechanisms, such as combining Diffusion Transformers (DiT) with CLIP-based image conditioning (37) to provide precise structural guidance during the translation process.

These broad innovations in general I2I methods lay a foundation for virtual staining, including techniques for maintaining image consistency under weak supervision, modeling data distributions and conditional probability distributions, as well as learning disentangled representations of content and style. However, a fundamental distinction exists in their underlying optimization objectives. General I2I methods primarily optimize for perceptual realism, global aesthetic coherence, and stylistic diversity. In contrast, histopathological translation is a biomedical mapping task where biological correctness absolutely supersedes pure visual appeal. The style transfer of pathological images must strictly preserve key diagnostic structures such as epithelial layers and nuclear atypia to avoid false cell population generation or distortion of important tissue features (38).

### 2.3 Virtual staining

Virtual Staining is an important branch of computational pathology aimed at converting unlabeled or conventional-stained images into images that simulate specific staining effects, such as IHC, special staining, or fluorescent-labeled images (17), through deep learning models to obtain molecular-level information (18, 39) without actual experiments. The technique not only significantly reduces detection costs and time, but also promotes the standardization of remote pathology and primary diagnosis. Early research focused mainly on single staining generation, such as H&E staining from autofluorescence images (29). In recent years, research has focused on the virtual generation of functional staining, especially key clinical markers such as p16, Ki-67, ER, HER2. Klockner et al. (40) Systematically reviewed the application of multiple deep generative models in virtual staining of breast cancer, achieving precise conversion from H&E images to IHC HER2, ER, PgR, and Ki-67 images. It provides a reliable technical pathway and open evaluation benchmark for low-cost, rapid auxiliary diagnosis of breast cancer biomarkers. Aggarwal et al. (41) developed the AI-based virtual staining platform VISTA, which can generate CD163+ virtual IHC images with high precision from H&E images and accurately identify M2 tumor-associated macrophages in HPV+ oropharyngeal squamous cell carcinoma. It provides an expandable computational pathological tool for tumor microenvironment assessment without additional experiments. Yan et al. (42) proposed DUST, a multifunctional virtual staining framework based on diffusion models, which achieves high-fidelity conversion among various staining modes such as H&E, Masson tri-color, PAS, and PASM through a dual-coding strategy and dynamic dual-output head design. However, these methods are mostly based on supervised learning and rely on perfectly aligned paired data, which is difficult to prepare. In contrast, weakly supervised or unsupervised methods are more practical. Guan et al. (43) proposed a virtual IHC staining model for breast cancer based on a generative adversarial network, which achieves high-fidelity, pixel-level aligned H&E to IHC staining conversion by introducing a dual-supervised information mining mechanism of optimal transport and pathological correlation, taking full advantage of the weak pairing relationship between H&E and adjacent layers of IHC sections. It significantly improves the accuracy and clinical applicability of virtual staining in the analysis of breast cancer whole section images. Yoon et al. (44) developed a deep learn-based interconnection framework that enables the conversion of unlabeled photoacoustic histological images to virtual H&E staining, feature extraction, and liver cancer (45) classification through interpretable virtual staining, U-Net segmentation, and step-based feature fusion classification, significantly enhancing the clinical applicability of unlabeled (46) imaging in digital pathology. The Prompt-Driven Universal Model (PD-UniST) (47) proposes a unified framework for unpaired H&E-to-IHC translation, dynamically guiding spatial localization and style decoupling through text prompts. To tackle the misalignment in realistic datasets, topology-aware pathological consistency matching (48) has been proposed to rigorously preserve the underlying tissue topological structures during weakly-paired IHC virtual staining. In the realm of diffusion models, D-VST (49) introduces a Diffusion Transformer to achieve pathology-correct and tone-controllable cross-dye translation on WSIs. Furthermore, a novel dual-path prompted inversion method (50) enables unpaired multi-domain histopathology virtual staining by utilizing visual prompts to independently control content and style, completely bypassing the need for aligned annotations.

For virtual stain transfer to achieve high clinical precision, it needs to extract robust pathology-aware structural and semantic information from the corresponding supervision signals. However, most existing virtual staining approaches emphasize either pixel-level alignment or generative diversity, and only implicitly consider diagnostic relevance. Alternatively, they completely rely on extracting supervised information from strictly paired spatial data, which significantly increases the costs of data collection and annotation. Few methods explicitly integrate lesion-level semantic supervision or pathology-oriented structural modeling into the generative process, particularly for cervical intraepithelial neoplasia grading tasks.

In this work, we differ from prior studies by incorporating a multi-instance learning–based auxiliary classification branch—thereby effectively utilizing bag-level weak supervision signals—and an attention-guided feature modulation strategy, which explicitly enforce lesion-aware semantic consistency during virtual IHC generation.

## 3 Methods

### 3.1 Overview

The proposed framework aims to translate H&E WSIs into virtual IHC images corresponding to p16 and Ki-67 staining while preserving diagnostically relevant morphological cues. To achieve this objective, we build upon the CycleGAN backbone due to its ability to learn unpaired cross-domain mappings, but extend it with three key innovations designed specifically for computational pathology. It is worth noting that although CycleGAN is originally designed for unpaired image-to-image translation, the proposed framework adopts a weakly paired training strategy. Specifically, whole-slide image registration is first performed to spatially align H&E and corresponding IHC slides. The aligned regions are then partitioned into patch-level pairs for training. However, due to inevitable tissue deformation, sectioning differences, and staining variability between adjacent slices, the resulting pairs are not strictly pixel-aligned. Therefore, the training paradigm in this study is considered weakly paired rather than fully paired, allowing the model to leverage structural correspondence while maintaining robustness to spatial misalignment.

#### 3.1.1 Plain–global attention block (P–G block)

A lightweight hybrid attention unit that augments the generator’s ability to jointly model fine-grained nuclear textures and long-range epithelial patterns. This is crucial for mimicking biomarker distribution, which is often defined by region-level patterns (e.g., basal expansion, block positivity) rather than pixel-level color transfer.

#### 3.1.2 MIL-based auxiliary classification branch

Inspired by the lesion-level diagnostic workflow, we introduce a MIL classifier that receives generated IHC patches and outputs CIN-related categories. This provides semantic constraints that force the generator to retain discriminative lesion characteristics rather than only focusing on superficial stain appearance.

#### 3.1.3 Attention-guided loss reweighting

To ensure that clinically meaningful structures (e.g., basal layers, atypical nuclei, mitotic figures) receive stronger supervision, the instance-level attention weights learned by the MIL network are used to spatially modulate cycle-consistency and perceptual losses.

**Figure 1:**
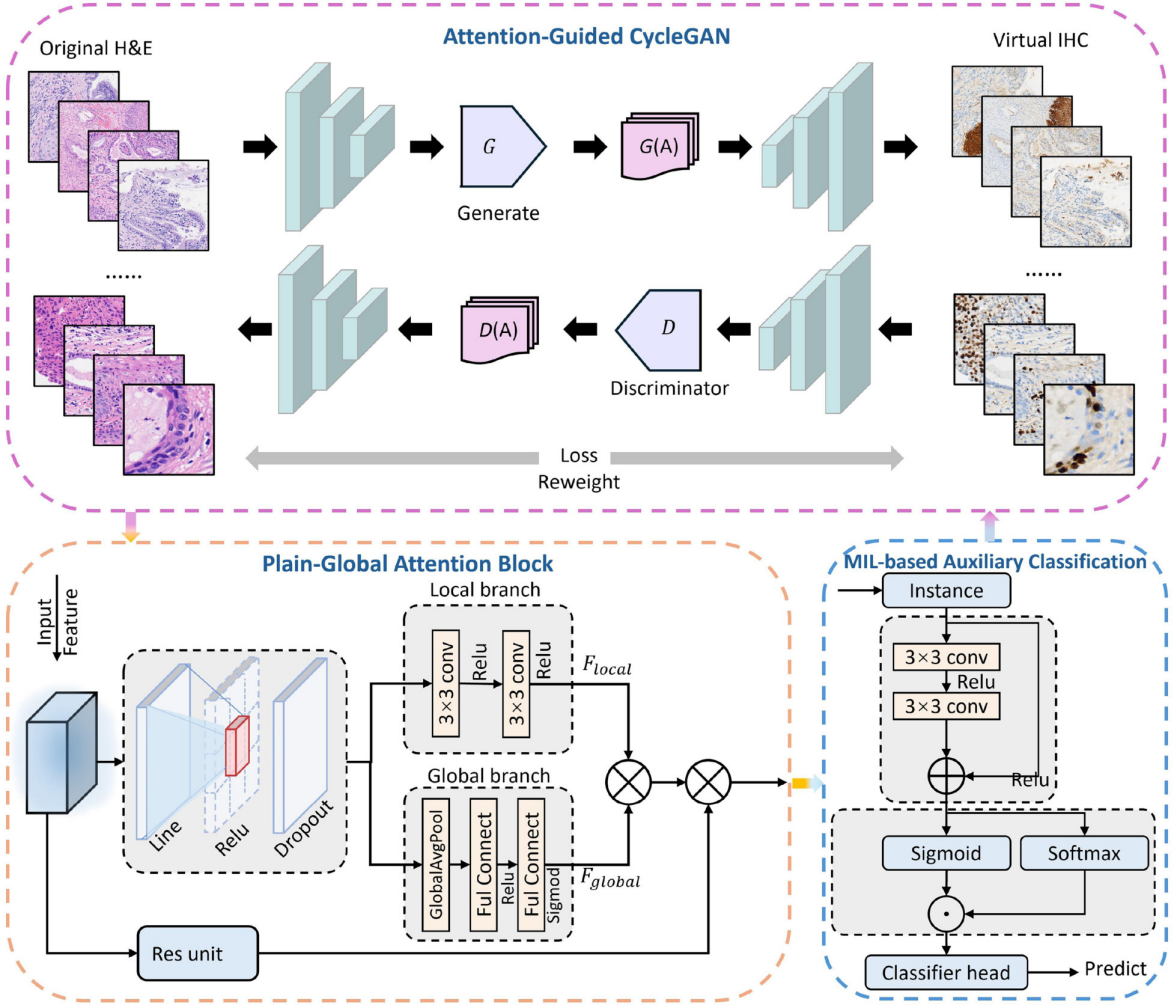
Detailed display of the method process.

Together, these components create a pathology-oriented virtual staining system that integrates structural preservation, semantic consistency, and biomarker plausibility. The overall architecture is illustrated in Figure 1.

### 3.2 Plain-global attention block

Virtual IHC generation requires the simultaneous preservation of cellular morphology (nuclei, chromatin, cytoplasm) and global tissue organization (epithelial stratification, basal expansion patterns). Conventional CycleGAN convolutional blocks may lose long-range contextual information, particularly in deeper layers. To address this limitation, we design the Plain–Global Attention Block, which fuses local features from shallow convolutions, global features from context-aware pooling, and adaptive attention to balance local and global cues. Given an input feature map $X\in R^{C\times H\times W}$, the module operates in three stages.

#### 3.2.1 Step 1: local feature extraction

The block begins by applying two cascaded 3×3 convolutions to capture high-frequency textures, including nuclear contours, chromatin granularity, and cytoplasmic boundaries as shown in Equation 1:

$$X_{\text{local}}=\phi \left(\mathrm{BN}\left({\mathrm{Conv}}_{3\times 3}\left(\mathrm{ReLU}\left({\mathrm{Conv}}_{3\times 3}(X)\right)\right)\right)\right)$$ (1)

where, BN() represents the batch normalization layer, which is used to accelerate convergence and stabilize training. ϕ is the normalization operation.

This step retains essential structural details that are necessary for accurate biomarker simulation.

#### 3.2.2 Step 2: global context aggregation

Global IHC expression patterns often correlate with epithelial depth (e.g., p16 block positivity from basal to mid-layer). Therefore, global context is essential. We generate two complementary descriptors: global Average Pooling (GAP) representing overall intensity and tissue composition; global Max Pooling (GMP) capturing the most salient regions as shown in Equation 2. We combine them:

$$g=\alpha \cdot \mathrm{GAP}(X_{\text{local}})+(1-\alpha )\cdot \mathrm{GMP}(X_{\text{local}})$$ (2)

where, α is the fusion coefficient, which controls the relative proportion of average and maximum pooling contributions. GAP is global average pooling, and GMP is global max pooling.

Nonlinear transformation via two fully connected layers produces an enhanced global descriptor as shown in Equation 3:

$$\tilde{g}=\sigma \left(W_{2}\;\delta (W_{1}g)\right)$$ (3)

where, δ() is the activation function of the first layer, and σ() is the activation function of the second layer.

And restored spatially via a 1×1 convolution and broadcasting as shown in Equation 4:

$$X_{\text{global}}=\mathrm{Expand}\left({\mathrm{Conv}}_{1\times 1}(\tilde{g})\right)$$ (4)

where, Expand() broadcasts ${\mathrm{Conv}}_{1\times 1}$ as ${\mathrm{Conv}}_{H\times W}$, maintaining the same spatial size as $X_{global}$ for element by element fusion.

#### 3.2.3 Step 3: feature fusion

Local and global signals are fused with an attention gate as shown in Equations 5 and 6: ?>

$$A=\sigma \left({\mathrm{Conv}}_{1\times 1}\left(X_{\text{local}}⊕\mathrm{Broadcast}(X_{\text{global}})\right)\right)$$ (5)

$$X_{\text{\textbackslash{},fuse}}=A⊙X_{\text{local}}+(1-A)⊙\mathrm{Broadcast}(X_{\text{global}})$$ (6)

where, ⊕ is the channel concatenation operation, ⊙ is multiplying by element points.

Finally, a residual connection stabilizes training and enriches representational depth as shown in Equation 7:

$$X_{\text{out}}={\mathrm{Conv}}_{3\times 3}(X_{\text{\textbackslash{},fuse}}+{\mathrm{Conv}}_{1\times 1}(X_{\text{local}}))+X$$ (7)

The block ensures both micro-level morphology and macro-level epithelial architecture are faithfully retained during the H&E-to-IHC transformation. This block is introduced to enhance feature representation in the generator network. Unlike commonly used attention modules such as squeeze-and-excitation (SE) or CBAM, which mainly focus on channel-wise or spatial feature reweighting, the block explicitly separates local feature extraction and global contextual modeling. Local convolutional operations capture fine-grained cellular morphology such as nuclear contours and chromatin texture, while the global branch aggregates long-range contextual information related to epithelial architecture. The two representations are then adaptively fused through an attention mechanism, enabling the network to simultaneously preserve cellular-level details and tissue-level structural patterns during virtual staining generation.

### 3.3 MIL-based auxiliary classification

In real histopathological diagnosis, lesion classification depends not only on individual nuclei but also on region-level patterns. Therefore, a naive image-to-image translation model may produce visually plausible but diagnostically misleading virtual stains.

To address this, we incorporate an MIL network to enforce lesion-aware semantic consistency.

#### 3.3.1 Input and construction

Each generated IHC patch (1024×1024 ) is fed into the MIL classifier. The network extracts instance-level features and aggregates them through an attention pooling mechanism that mimics the diagnostic focus of human pathologists.

Let $x_{t}$ denote the generated patch. The MIL network outputs class logits based on learned instance-level representations aggregated through attention pooling. An auxiliary classification branch based on multiple instance learning (MIL) is introduced to guide the training process. In this framework, slide-level CIN labels are used as coarse-grained supervision signals, while individual image patches are treated as instances within a bag under the MIL assumption. Patch-level predictions are therefore learned in a weakly supervised manner without explicit instance annotations. The auxiliary classification loss encourages the generator to maintain lesion-related structural features during the H&E-to-IHC translation process.

#### 3.3.2 Auxiliary classification loss

The auxiliary classification objective encourages the generator to retain discriminative features relevant to CIN grading.

Given the MIL output as shown in Equation 8, 9, and 10: ?>

$$z=\left(W_{\text{aux}}\;h+b_{\text{aux}}\right)/\tau$$ (8)

$$y_{c}=\mathrm{Softmax}(z_{c})=\frac{e^{z_{c}}}{\sum_{k=1}^{K}e^{z_{k}}}$$ (9)

where h is the aggregated representation and τ is a factor controlling confidence.

The loss is defined as

$$L_{\text{MIL-aux}}=-\sum_{c=1}^{K}\omega_{c}\;y_{c}^{\left(\text{gt}\right)}\log y_{c}+\lambda \|W_{\text{aux}}{\|}_{2}^{2}$$ (10)

This loss forces the generator to preserve lesion-specific cues relevant to p16/Ki-67 interpretation—such as basal hyperactivity, nuclear overlapping, and epithelial disorganization.

### 3.4 Attention-guided loss reweighting

Traditional CycleGAN loss functions treat all regions equally. However, CIN diagnosis relies heavily on specific regions: the transformation zone, basal and parabasal layers, regions with abnormal proliferation, and block-positive or sharply localized staining zones as shown in Equations 11 and 12.

For cycle-consistency:

$$L_{\text{cycle}}=\lambda_{\text{cyc}}\left(\alpha L_{\text{cycle}}^{\text{\textbackslash{},forward}}+(1-\alpha )L_{\text{cycle}}^{\text{backward}}\right)$$ (11)

where high-attention regions receive stronger supervision.

For perceptual similarity across L layers:

$$L_{\text{\textbackslash{},perc}}=\lambda_{\text{\textbackslash{},perc}}\sum_{l=1}^{L}\beta_{l}\|\phi_{l}(X)-\phi_{l}(\hat{X}){\|}_{2}^{2}$$ (12)

This ensures perceptual similarity specifically in diagnostically important regions. Regions marked as diagnostically important by the MIL module receive stronger gradients, helping maintain staining patterns consistent with real IHC expressions. The clinically relevant regions are identified through instance-level attention weights generated by the MIL-based auxiliary classification branch. These attention scores indicate the relative contribution of each patch to the slide-level CIN prediction and therefore highlight diagnostically informative regions. During training, the attention weights are used to spatially reweight the cycle-consistency and perceptual losses, assigning larger weights to regions associated with abnormal epithelial structures while reducing the influence of less informative background areas.

### 3.5 Overall objective function

The total loss for training the generator is formulated as shown in Equation 13

$$L_{\text{total}}=L_{\text{adv}}+\lambda_{\text{cyc}}L_{\text{cycle}}+\lambda_{\text{id}}L_{\text{id}}+\lambda_{\text{\textbackslash{},perc}}L_{\text{\textbackslash{},perc}}+\lambda_{\text{MIL}}L_{\text{MIL-aux}}$$ (13)

where $\lambda_{\text{cyc}},\lambda_{\text{id}},\lambda_{\text{\textbackslash{},perc}},\lambda_{\text{MIL}}$ are weighting coefficients.

This combination allows the model to balance image realism, structural preservation, perceptual accuracy, and lesion-aware semantics during training, leading to more reliable virtual IHC outputs. The perceptual loss employs a pre-trained VGG-16 network as the feature extractor. we compute the multi-level perceptual distances using the feature maps extracted from five intermediate layers corresponding to the five spatial resolution blocks. The perceptual similarity of L layers is defined as:

In order to demonstrate the workflow of the framework more clearly, its pseudocode is shown in Algorithm 1.


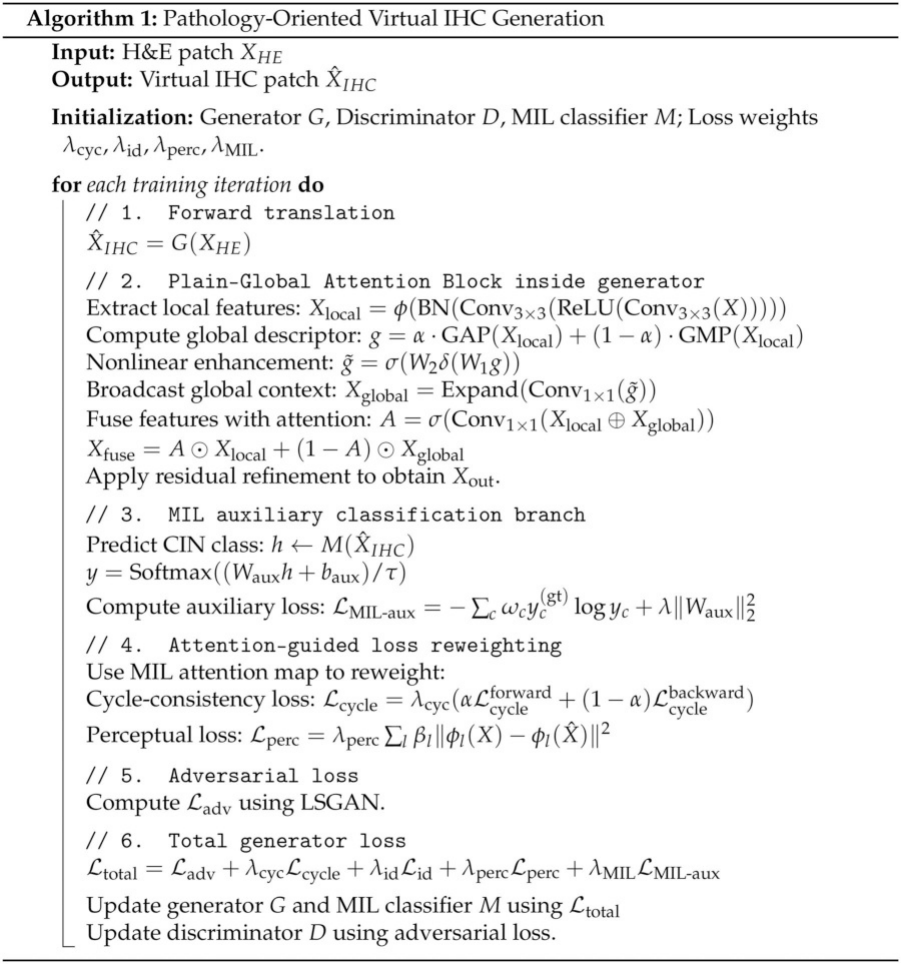


## 4 Experiment

### 4.1 Dataset

This study retrospectively collected H&E and corresponding p16/Ki-67 IHC whole-slide images from patients diagnosed with cervical squamous intraepithelial lesions at Ganzhou Maternal and Child Health Hospital between January 2022 and December 2024. Eligible participants were women aged 18–65 years with histopathologically confirmed CIN1, CIN2, or CIN3, and consecutive H&E and IHC sections available from paraffin-embedded blocks. Patients with a history of cervical surgery, pregnancy, autoimmune disease, or systemic immunosuppression were excluded. A total of 60 patients meeting the criteria were included—20 for each CIN grade—together with 20 chronic cervicitis cases serving as non-neoplastic controls. The classification of cervical lesions follows the standard Cervical Intraepithelial Neoplasia (CIN) grading system, which categorizes biopsies into three stages(CIN1, CIN2, CIN3) based on the extent of cellular atypia within the epithelial layers. Additionally, cases exhibiting non-precancerous inflammatory changes are classified as a distinct, separate category.

All slides were prepared following standard clinical histopathology procedures. Serial 4-μm sections were cut to ensure structural correspondence between H&E and IHC slides. Digital scanning was performed using the same whole-slide scanner at 40× magnification (0.25 μm/pixel) under unified color calibration settings to minimize device-related variability. Before patch extraction, all WSIs underwent stain normalization. From each WSI pair, pathologists identified regions of diagnostic relevance, including transformation zones, epithelial stratification layers, and atypical proliferative regions. Corresponding image fields were cropped from the serial sections to create paired H&E/IHC patches of 1024×1024 pixels. These diagnostically relevant regions are manually annotated by expert pathologists prior to patch extraction. Background-dominant tiles were removed automatically using tissue-mask filtering. The dataset was randomly divided at the patient level with a 7:1:2 split for training, validation, and testing, ensuring that samples from the same individual did not appear in multiple partitions. After quality control and background filtering, a total of 8,640 paired H&E/IHC patches (1024×1024 pixels) were obtained. Among them, 6,048 patches were used for training, 864 patches for validation, and 1,728 patches for testing. The patient-level partition strategy effectively prevented data leakage and ensured independence across subsets, thereby improving the reliability and generalizability assessment of the proposed framework.

To further evaluate the generalization capability of the proposed model, we additionally introduce the publicly available ACROBAT dataset as an external validation dataset. The ACROBAT dataset contains 4,212 whole-slide images (WSIs) from 1,153 patients, including paired H&E and IHC-stained slides with biomarkers such as Ki-67, HER2, ER, and PgR. All data are collected from routine clinical workflows, ensuring realistic variability in staining conditions, tissue morphology, and scanning protocols. It should be noted that the ACROBAT dataset is not specifically aligned with cervical pathology tasks. Instead, it is introduced to evaluate the cross-domain generalization capability of the proposed model under heterogeneous data distributions, including variations in tissue types, staining protocols, and acquisition conditions. Therefore, this dataset serves as an external robustness benchmark rather than a task-specific validation set.

To construct a training-ready dataset, we first perform WSI-level registration using the open-source toolkit VALIS, followed by manually quality control to remove poorly aligned regions. The aligned WSIs are then divided into paired patches of size 256×256 pixels. The dataset is split into training, validation, and test sets according to the official protocol.

### 4.2 Experimental setup

All experiments were conducted on an NVIDIA RTX 3090 GPU (24 GB memory) using PyTorch as the implementation framework. The model was trained end-to-end using the Adam optimizer with an initial learning rate of 1e-4. The batch size was set to 4 due to GPU memory constraints.To ensure stable convergence, a step-wise learning rate decay schedule was adopted, with the learning rate reduced at epochs 30, 90, and 150. The training process was performed for a total of 200 epochs.To further stabilize adversarial training, the discriminator was equipped with spectral normalization. In addition, gradient penalty and gradient clipping were applied during optimization to prevent gradient explosion and improve training stability.

During training, we applied commonly used histopathology augmentations—including random rotations, flips, color perturbations, mild blurring, and stain-intensity variation—to enhance robustness to morphological and staining variations. Augmentations were restricted to the H&E domain to prevent label leakage. All hyperparameters were kept fixed across experiments to ensure comparability. The selection of model architecture components and training strategies was guided by pathological diagnostic principles, aiming to balance image realism with structural and semantic fidelity rather than purely optimizing visual appearance. The proposed framework contains approximately 32.6 million trainable parameters, including the generator, discriminator, and MIL auxiliary branch. Under the RTX 3090 GPU (24 GB memory) environment, the average inference time is approximately 118 ms per 1024×1024 patch during testing (batch size = 1). For a typical whole-slide image containing 400–600 diagnostic patches, the end-to-end inference time is approximately 50–70 s, excluding slide loading and preprocessing. These results indicate that the model achieves a favorable balance between computational complexity and practical deployability in digital pathology workflows.

### 4.3 Overall effect display

**Figure 2:**
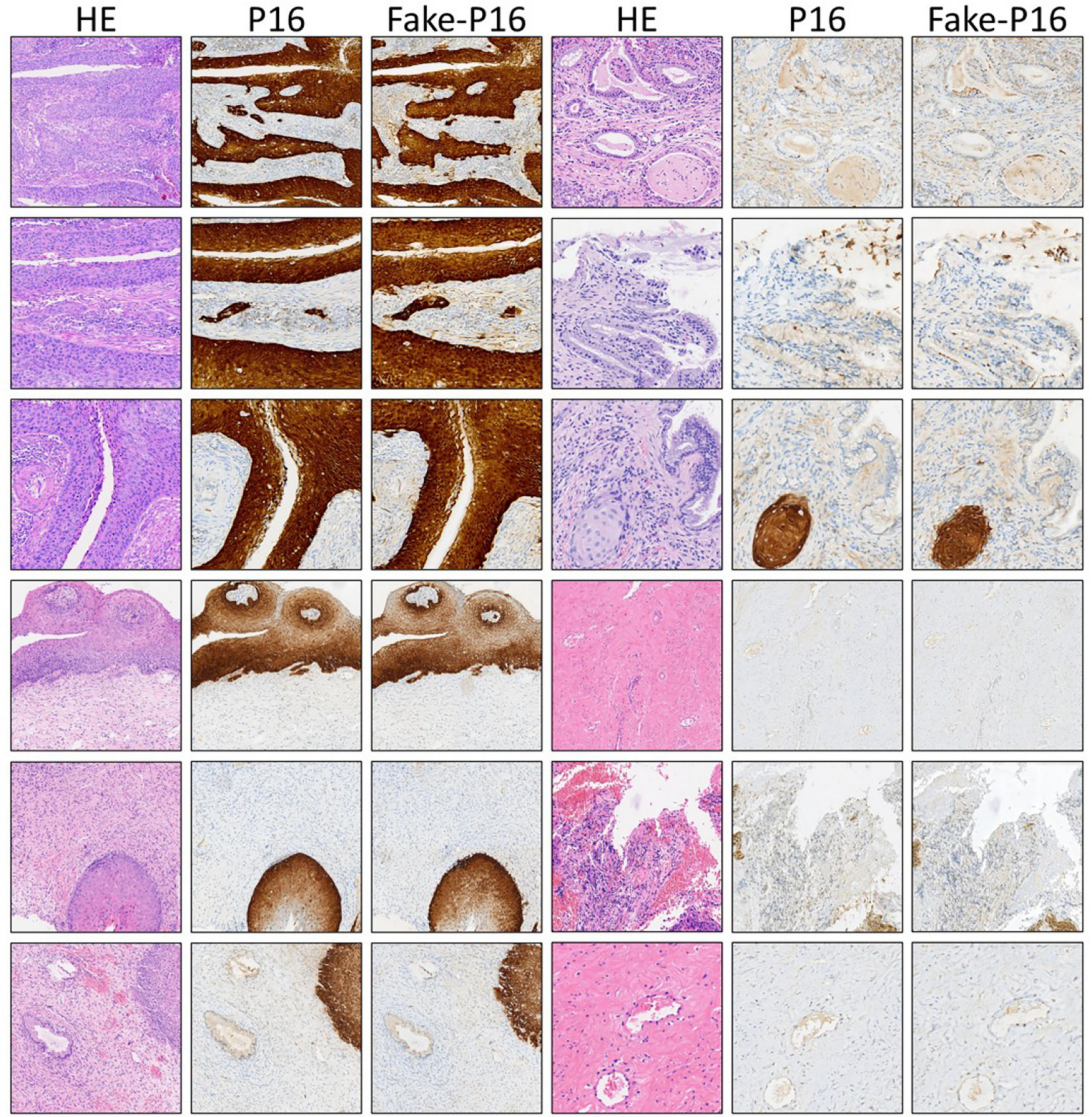
P16 virtual dyeing conversion results.

**Figure 3:**
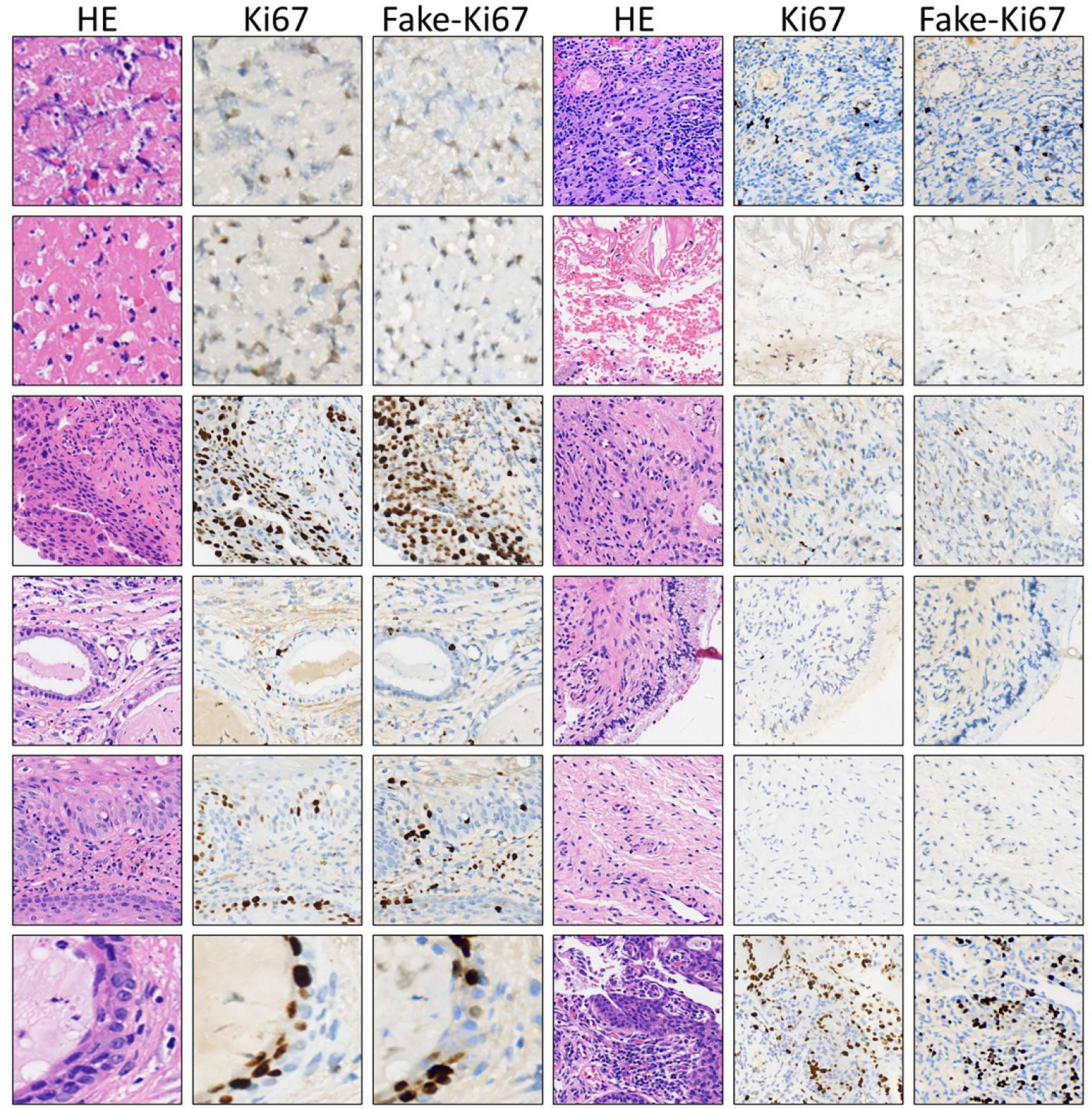
Ki67 virtual dyeing conversion results.

As shown in Figure 2, the generated p16 virtual staining reproduces characteristic block-type positivity patterns observed in high-grade lesions. Similarly, Figure 3 demonstrates that the Ki-67 virtual staining accurately reflects proliferative expansion across epithelial layers. The synthesized images closely mimic the visual characteristics of real IHC slides, including chromatin detail, epithelial layer integrity, nuclear morphology, and the expected basal-to-superficial distribution of positive staining. The model successfully replicates block-type positivity in p16 and proliferative-layer expansion in Ki-67, which are critical indicators in CIN grading. Fine-grained textures such as nuclear contours and chromogen patterns are also preserved, demonstrating the model’s ability to capture clinically meaningful structural cues rather than merely color appearance.

In addition, the generated virtual staining demonstrates good specificity by selectively highlighting lesion-relevant epithelial regions while largely suppressing non-epithelial background tissue. Consistent qualitative patterns and stable quantitative metrics across validation folds further suggest reasonable robustness under variations in staining appearance and tissue morphology.

The quantitative results summarized in Table 1 corroborate these visual observations. On the test set, p16 and Ki-67 virtual stains achieved FID scores of 28.4 and 31.2, indicating strong overall realism. It is worth noting that the Fréchet Inception Distance (FID), originally developed for natural image generation tasks, may not fully capture the domain-specific characteristics of histopathology images, such as fine-grained cellular morphology and staining-specific patterns. Nevertheless, FID has been widely adopted in recent virtual staining and medical image synthesis studies as a general measure of distribution alignment between generated and real images. In this work, we use FID as a complementary metric to assess global visual realism and distribution consistency.Meanwhile, Cohen’s kappa is used to measure the agreement between predicted labels and reference labels while accounting for chance agreement. It is defined as: $\kappa =\frac{\;p_{o}-p_{e}}{1-p_{e}}$, where $p_{o}$ denotes the observed agreement and $p_{e}$ represents the expected agreement by random chance. A higher kappa value indicates better consistency between predictions and ground truth. The corresponding kappa values (0.76 and 0.72) demonstrate high diagnostic consistency with labels inferred from real IHC. Meanwhile, SSIM and LPIPS remain stable across biomarkers, and the HIST scores suggest good alignment in global color/texture distribution. Together, these results confirm that the proposed model achieves a balanced improvement in perceptual quality, structural preservation, and diagnostic reliability.

**Table 1:** Comprehensive performance display of P16 and Ki67.

Index	FID	Kappa	SSIM	LPIPS	HIST
P16	28.4±0.72	0.76±0.061	0.812±0.053	0.371±0.044	0.830±0.037
Ki67	31.2±0.74	0.72±0.042	0.798±0.045	0.362±0.036	0.842±0.038

### 4.4 Comparative experiments

**Figure 4:**
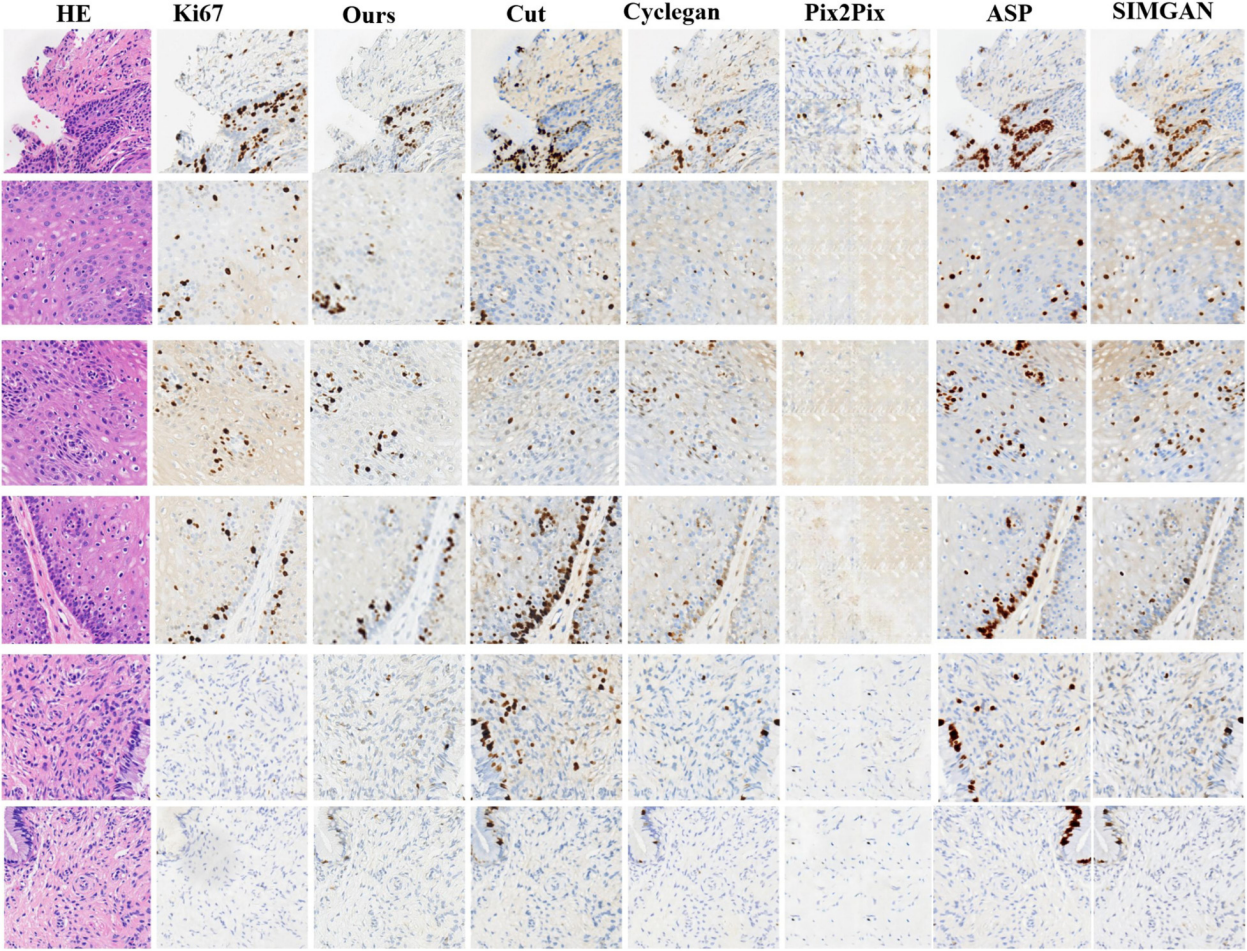
Comparative experiments results.

To further evaluate the effectiveness of the proposed method, we compared it with several widely used image-to-image translation frameworks, including CycleGAN, CUT, Pix2Pix, ASP, and SIMGAN. All baseline models were trained under exactly the same optimization settings, dataset partitions, and pre-processing pipelines to ensure fairness. Qualitative results in Figure 4 reveal that conventional GAN-based methods tend to produce either over-smoothed textures or irregular stain distributions, whereas our method yields more faithful nuclear morphology and more stable chromogen expression patterns.

The quantitative comparison in Table 2 demonstrates consistent superiority across all metrics. Our method achieved the lowest FID, indicating stronger visual realism, and the highest kappa, reflecting improved diagnostic consistency. SSIM and HIST values further show superior structural and color fidelity. The lowest LPIPS score suggests that the perceptual distance between virtual and real IHC stains is also minimized. These findings confirm that the combination of global attention, MIL-guided semantic constraints, and weighted feature modulation contributes substantially to translation quality. These quantitative improvements are consistent with the qualitative comparisons illustrated in Figure 4, indicating that the proposed method achieves both visual and diagnostic advantages.

**Table 2:** Comparative experimental performance display.

Index	Performance				
Model	FID	Kappa	SSIM	LPIPS	HIST
Ours	29.1±0.77	0.752±0.032	0.807±0.044	0.379±0.036	0.827±0.038
Cut	45.6±1.31	0.634±0.043	0.694±0.045	0.413±0.041	0.744±0.043
Cyclegan	54.8±1.42	0.582±0.061	0.658±0.074	0.407±0.066	0.765±0.072
Pix2Pix	65.3±1.54	0.528±0.046	0.623±0.071	0.391±0.076	0.787±0.073
ASP	42.6±1.58	0.681±0.046	0.706±0.048	0.406±0.077	0.811±0.046
SIMGAN	33.5±1.13	0.658±0.037	0.643±0.082	0.388±0.024	0.769±0.057

### 4.5 Ablation experiments

**Figure 5:**
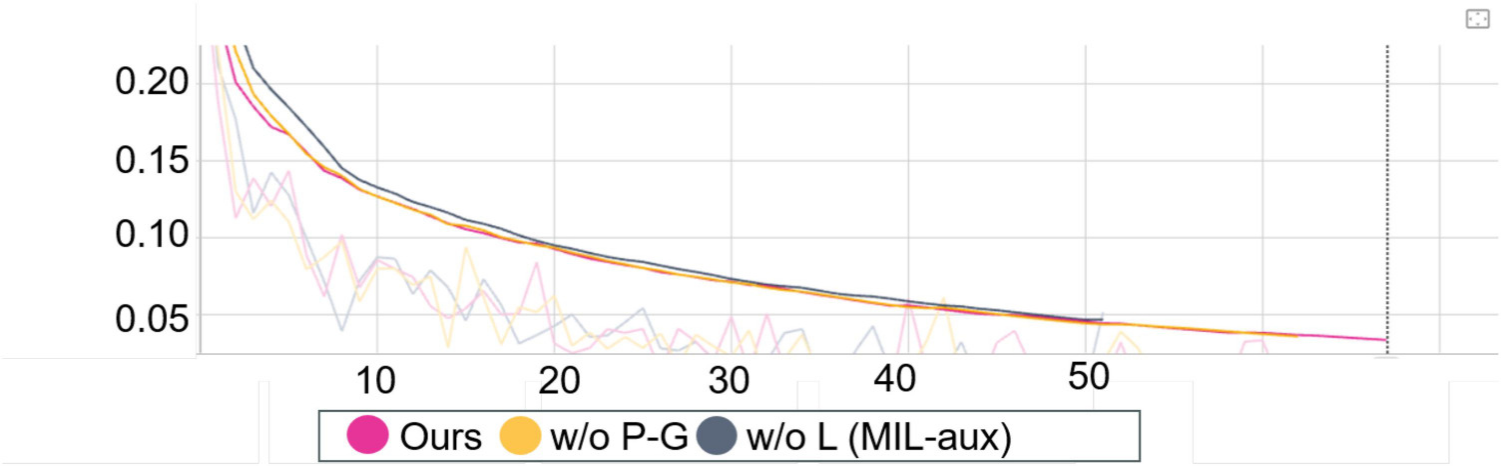
Ablation experimental training curves.

To investigate the role of each key module, ablation experiments were conducted by removing the Plain–Global Attention Block or the MIL-based auxiliary loss. As shown in Figure 5, excluding the attention block results in noticeable degradation of long-range structural coherence, leading to weaker nuclear boundaries and less uniform stain distribution. Similarly, removing the MIL constraint diminishes lesion-related semantic cues, producing virtual stains that appear visually acceptable but exhibit reduced diagnostic consistency.

**Figure 6:**
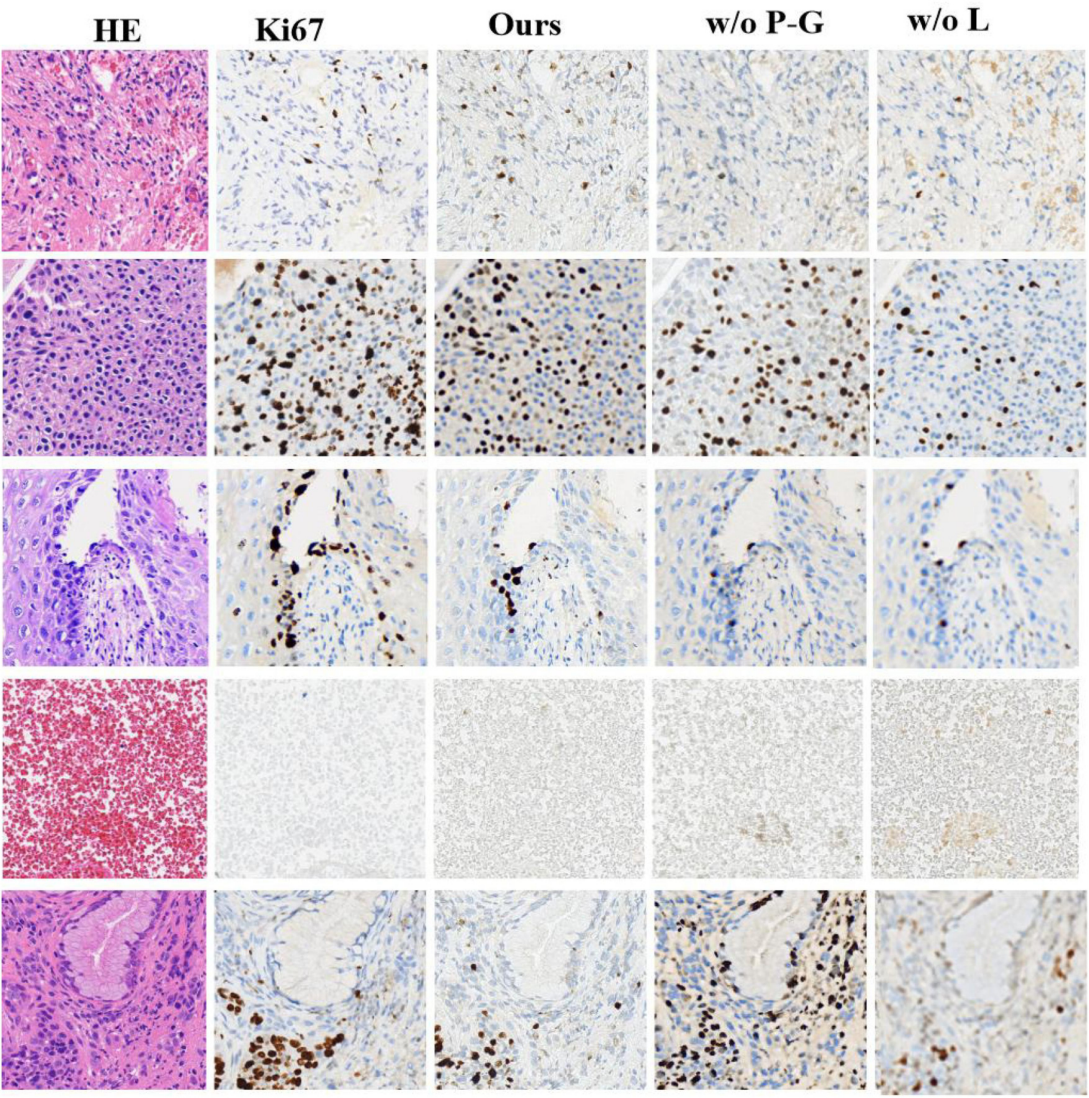
Ablation experiments results.

The ablation experiment training curve shown in Figure 6 also indicates that the proposed complete architecture achieves faster and more stable convergence than individual parts.

The ablation results summarized in Table 3 confirm these tendencies. Removing the attention block significantly increases FID and reduces SSIM, indicating a loss of global structure and textural integrity. Excluding the MIL-auxiliary loss leads to a clear drop in kappa and HIST, demonstrating that semantic supervision is crucial for maintaining biomarker-dependent staining patterns. Overall, the full model provides the best performance across all evaluation metrics, demonstrating that both components play complementary roles in enhancing visual fidelity and diagnostic relevance.

**Table 3:** Ablation experiment performance demonstration.

Index	FID	Kappa	SSIM	LPIPS	HIST
Ours	29.0±0.73	0.76±0.031	0.815±0.042	0.377±0.061	0.831±0.068
w/o P-G	37.1±1.12	0.71±0.029	0.768±0.049	0.412±0.043	0.829±0.067
(w/o $L_{\text{MIL-aux}}$)	34.6±1.31	0.68±0.027	0.742±0.047	0.42±0.067	0.817±0.072

### 4.6 Comparison with standard attention modules

To further validate the effectiveness of the proposed Plain–Global Attention (P–G) block, we compare it with several widely used attention mechanisms, including Squeeze-and-Excitation (SE), Convolutional Block Attention Module (CBAM), and self-attention (Non-local block).

For fair comparison, we replace the proposed P–G block in the generator with each baseline attention module while keeping all other network components and training settings unchanged.

The following table summarizes the quantitative results. It can be observed that although all attention mechanisms improve the baseline CycleGAN to some extent, the proposed P–G block consistently achieves the best performance across all evaluation metrics. In particular, it yields the lowest FID score and the highest Kappa and SSIM values, indicating superior image realism, structural fidelity, and diagnostic consistency.

The SE module mainly focuses on channel-wise feature recalibration, which lacks spatial awareness. CBAM introduces both channel and spatial attention but still relies on sequential attention refinement, which may not fully capture long-range contextual dependencies. The self-attention mechanism models global interactions but often introduces high computational cost and may weaken fine-grained local details.

In contrast, the proposed P–G block explicitly decouples local feature extraction and global context modeling, and adaptively fuses them through an attention gate. This design enables the model to simultaneously preserve cellular-level morphology and tissue-level structural patterns, which are critical for virtual IHC generation.

These results demonstrate that the proposed attention mechanism is better aligned with the pathological characteristics of the task and provides a more effective inductive bias than generic attention modules (Table 4).

**Table 4:** Comparison with different attention mechanisms.

Attention type	FID	Kappa	SSIM	LPIPS	HIST
None (Baseline)	41.3±1.21	0.662±0.045	0.721±0.052	0.421±0.038	0.781±0.044
SE	36.8±1.08	0.701±0.037	0.754±0.048	0.402±0.035	0.803±0.039
CBAM	34.5±0.97	0.718±0.033	0.768±0.041	0.394±0.032	0.812±0.036
Self-Attention	33.7±1.02	0.726±0.031	0.774±0.045	0.389±0.034	0.816±0.038
**P–G (Ours)**	** 29.0±0.73 **	** 0.760±0.031 **	** 0.815±0.042 **	** 0.377±0.061 **	** 0.831±0.068 **

Bold values are used to highlight the performance of our proposed method.

### 4.7 Auxiliary diagnostic evaluation experiments

To further validate the effectiveness of the proposed virtual staining of p16 and Ki-67 for auxiliary diagnosis, we design a simulated diagnostic experiment. Specifically, we adopt a standard Attention-based Multiple Instance Learning (ABMIL) framework to perform whole-slide image (WSI)-level CIN grading. In this setting, the generated virtual p16 and Ki-67 images are combined with the original H&E images as multi-modal inputs. Feature-level fusion is implemented using a standard concatenation strategy, where patch embeddings from different modalities are extracted and concated in feature channel. To assess the contribution of virtual staining, we compare the proposed multi-modal setting (H&E + virtual p16 + virtual ki67) against a baseline model that uses only H&E images as input. We use ROC-AUC, Accuracy, Sensitivity, Specificity as evaluation metrics.

The results of the auxiliary diagnostic evaluation experiment demonstrate that the proposed virtual staining approach can significantly improve the classification performance of MIL model, as reflected by consistent gains in all evaluation metrics (Table 5). In addition, a reduction in performance variance indicates improved model stability. These findings suggest that incorporating virtual p16 and Ki-67 staining provides complementary diagnostic information beyond H&E morphology, thereby enhancing the effectiveness of MIL-based classification and supporting its role as an auxiliary diagnostic tool.

**Table 5:** Auxiliary diagnostic evaluation experiment performance demonstration.

Grading results	AUC	Accuracy	Sensitivity	Specificity
HE+Ki67+P16	0.863±0.038	0.730±0.031	0.697±0.037	0.763±0.040
HE only	0.814±0.045	0.667±0.053	0.645±0.034	0.689±0.050

### 4.8 Performance comparison on the external ACROBAT dataset

Table 6 presents the quantitative results on the external ACROBAT dataset. Due to the inherent domain shift between the internal dataset and ACROBAT—such as differences in tissue type, staining protocols, and image acquisition conditions—all methods exhibit a certain degree of performance degradation compared to in-domain evaluations. This observation is consistent with practical cross-institution scenarios and highlights the increased difficulty of generalization under heterogeneous data distributions.

**Table 6:** Performance comparison on the external ACROBAT dataset.

Index	Performance				
Model	FID	Kappa	SSIM	LPIPS	HIST
Ours	34.7±0.95	0.701±0.038	0.762±0.051	0.402±0.042	0.801±0.041
Cut	52.3±1.48	0.591±0.052	0.661±0.053	0.436±0.046	0.721±0.048
Cyclegan	60.8±1.67	0.548±0.065	0.629±0.081	0.429±0.071	0.738±0.079
Pix2Pix	71.6±1.82	0.497±0.058	0.598±0.083	0.415±0.082	0.762±0.081
ASP	48.9±1.63	0.642±0.051	0.676±0.056	0.428±0.081	0.783±0.052
SIMGAN	38.6±1.27	0.621±0.044	0.612±0.087	0.409±0.031	0.751±0.063

Despite these challenges, the proposed method consistently outperforms all competing approaches across all evaluation metrics. Specifically, our model achieves the lowest FID (34.7±0.95), indicating superior distribution alignment with real IHC images, while also obtaining the highest Kappa (0.701±0.038) and SSIM (0.762±0.051), demonstrating better structural consistency and diagnostic agreement. Compared with baseline methods such as CycleGAN and CUT, which show more significant performance degradation, our approach maintains more stable results under cross-domain conditions. These findings suggest that the proposed framework effectively captures pathology-relevant invariant features, leading to improved robustness and generalization capability for real-world clinical applications.

### 4.9 Expert pathologist evaluation

To further assess the clinical reliability of the proposed virtual staining method, we conducted an expert-based evaluation involving two board-certified pathologists with 10 and 15 years of diagnostic experience, respectively.

Each pathologist independently reviewed the same set of whole-slide images under two conditions: (1) H&E-only, and (2) H&E augmented with generated virtual p16 and Ki-67 staining. The evaluation was performed in a blinded manner, and the order of cases was randomized to minimize bias.

The diagnostic performance before and after introducing virtual staining was quantified in terms of accuracy, precision, and sensitivity. As shown in Table 7, the inclusion of virtual staining consistently improved diagnostic performance across all metrics, indicating that the generated IHC images provide clinically meaningful complementary information for CIN grading.

**Table 7:** Expert diagnostic performance before and after virtual staining.

Condition	Accuracy	Precision	Sensitivity
H&E only	0.682	0.665	0.648
H&E + Virtual IHC	0.746	0.732	0.715

## 5 Conclusion

Compared with conventional immunohistochemistry, which requires additional staining procedures, reagents, and laboratory infrastructure, the proposed virtual staining framework operates directly on routine H&E slides. This enables improved workflow efficiency and scalability, particularly in resource-limited settings. Rather than replacing traditional IHC, the proposed approach is intended as a complementary tool to support pathological assessment when conventional staining is unavailable or delayed.

The proposed framework demonstrates improved structural fidelity, perceptual quality, and diagnostic consistency.

This study presents a virtual IHC framework capable of generating p16- and Ki-67-like images directly from H&E-stained sections using an enhanced CycleGAN architecture. By incorporating lightweight attention mechanisms, MIL-derived semantic constraints, and attention-guided loss reweighting, the proposed method achieves high structural fidelity, strong perceptual similarity, and consistent diagnostic performance compared with real IHC staining.

The findings suggest that virtual IHC can effectively highlight molecular features relevant to CIN grading and may serve as a complementary tool to support pathological assessment in scenarios where traditional IHC is limited. The method provides a reproducible, cost-efficient, and scalable computational solution that has the potential to assist diagnostic decision-making and support clinical workflows, particularly in resource-constrained environments. In practical pathology workflows, the proposed method can be integrated as a pre-screening or decision-support tool, assisting pathologists in prioritizing suspicious regions, reducing unnecessary staining, and improving diagnostic efficiency.

To further evaluate clinical applicability, we incorporated an expert-based diagnostic assessment in which experienced pathologists independently reviewed H&E slides with and without virtual staining assistance. The observed improvements in diagnostic accuracy, precision, and sensitivity provide preliminary evidence that the generated virtual IHC images can offer clinically meaningful complementary information.

Despite the encouraging results, several limitations should be acknowledged. First, the dataset used in this study is relatively limited in size and originates from a single institution, which may restrict the generalizability of the trained model. Second, variations in staining protocols, scanner characteristics, and rare pathological patterns may introduce domain shifts when applying the model to data from different clinical centers. In addition, the manual selection of diagnostically relevant regions may introduce selection bias by emphasizing salient areas while underrepresenting the full variability of whole-slide images. To partially address this issue, we further evaluated the model on the ACROBAT dataset, which is derived from routine clinical workflows without targeted region selection. The consistent performance on this dataset suggests that the proposed method does not rely excessively on curated regions.

Furthermore, as virtual staining is inherently a predictive process, there exists a potential risk of false-positive or biologically inconsistent biomarker expression at the local level. In this work, we mitigate this issue by incorporating attention-guided supervision and MIL-based semantic constraints, which enforce consistency at the lesion and slide levels rather than relying solely on pixel-wise fidelity. In addition, the quantitative evaluation results, including diagnostic consistency metrics, together with expert assessment, provide indirect evidence supporting the biological plausibility of the generated staining patterns. Nevertheless, more rigorous validation, such as large-scale multi-center clinical studies and region-level biomarker verification, remains necessary.

Future work will focus on expanding multi-center datasets, improving domain generalization, and developing more reliable biological validation strategies to further enhance the robustness and clinical applicability of virtual staining methods.

## Data Availability

The original contributions presented in the study are included in the article/Supplementary Material, further inquiries can be directed to the corresponding author/s.
